# Association analysis of single nucleotide polymorphisms in the LIFR gene with lambing number in sheep

**DOI:** 10.3389/fvets.2025.1629162

**Published:** 2025-07-10

**Authors:** Yuliang Wen, Yuping Xiang, Runan Zhang, Kai Liu, Yufang Liu, Mingxing Chu

**Affiliations:** ^1^College of Animal Science and Veterinary Medicine, Henan Institute of Science and Technology, Xinxiang, China; ^2^State Key Laboratory of Animal Biotech Breeding, Institute of Animal Science, Chinese Academy of Agricultural Sciences, Beijing, China

**Keywords:** lambing number, LIFR, molecular marker, MassARRAY, sheep

## Abstract

Lambing number trait in sheep is a complex trait controlled by multiple genes, and low lambing number has a severe economic impact on the sheep industry. Previous studies have shown that Sparc/Osteonectin, cwcv, and kazal-like domain proteoglycan 1 (*SPOCK1*), A disintegrin and metalloproteinase with thrombospondin-like repeats 1 (*ADAMTS1*), heparin-binding epidermal growth factor-like growth factor (*HBEGF*), and leukemia inhibitory factor receptor (*LIFR*) are involved in mammalian reproduction. However, the effects of these genes on lambing number in sheep are still unclear. In this study, single nucleotide polymorphism (SNP) loci at the above four genes were genotyped in five sheep breeds (two single-born sheep breeds and three multiple-born sheep breeds with a total of 768 sheep) using the Sequenom MassARRAY^®^ SNP assay, and their associations with the lambing number in small-tailed Han sheep were analyzed. The results showed that a total of six SNP loci (c.^*^1633A>G, c.^*^1388T>C, c.^*^1095G>A, c.1847A>G, c.^*^2403A>G, and c.^*^127T>C) were identified in the four genes, *SPOCK1, ADAMTS1, HBEGF*, and *LIFR*. All the above SNPs had three genotypes in five sheep breeds. The population genetic analysis of SNPs of the four genes and the association analysis with lambing number showed that the polymorphism information content of the five sheep breeds of small-tailed Han sheep, Hu sheep, Cele black sheep, Sunite sheep, and Bamei mutton sheep was between 0 and 0.37, and a few sheep breeds were in Hardy-Weinberg equilibrium (p>0.05). The c.^*^127 T>C locus of the *LIFR* gene may be affected by natural or artificial selection in these sheep breeds. Additionally, the association analysis between the c.^*^127 T>C locus of *LIFR* gene and the lambing number of small-tailed Han sheep showed that the c.^*^127 T>C locus of the *LIFR* gene was significantly associated with the lambing number of the second, third, and average parity of smalltailed Han sheep (*p* < 0.05). The lambing number of ewes with the *LIFR* CC genotype was significantly lower than that with the TT and CT genotypes (*p* < 0.05). The *LIFR* protein interaction network was also predicted and found to interact with the reported ciliary neurotrophic factor (CNTF), cardiotrophinlike cytokine factor 1 (CLCF1), interleukin 6 cytokine family signal transducer (IL6ST), and ciliary neurotrophic factor receptor (CNTFR) proteins. In conclusion, the c.^*^127 T>C locus of *LIFR* gene can be used as a candidate genetic molecular marker to increase lambing numbers in polytocous sheep.

## 1 Introduction

Sheep are important economic animals as they provide valuable agricultural products to humans. Increasing the reproductive performance of sheep is one of the most effective ways to enhance the sheep industry and increase its economic benefits (1). Traditional breeding methods face challenges in rapidly increasing lambing numbers due to the low heritability. Compared to traditional breeding methods, molecular marker-assisted selection breeding methods significantly shorten the breeding cycle and improve the success rate of breeding (2). Therefore, molecular marker-assisted selection breeding is significant in improving the lambing number of sheep. In particular, a few studies have found that general transcription factor IIA subunit 1 (*GTF2A1*) (3), GATA binding protein 4 (*GATA4*) (4), growth differentiation factor 9 (*GDF9*) (5), mutations at loci such as prolactin receptor (*PRLR*), insulin like growth factor 1 (*IGF1*), and lymphoid enhancer-binding factor (*LEF*) (6) have been associated with reproductive traits in sheep, leading to differences in lambing numbers. Assisted selection using these molecular markers can improve the reproductive efficiency of sheep breeds, leading to higher productivity and improved economic returns.

Sparc/Osteonectin, cwcv, and kazal-like domain proteoglycan 1 (*SPOCK1*) gene inhibits the apoptosis of tumor cells and is located in the nucleus and mitochondria of tumor cells. However, the physiological function of this gene has not been determined (7). It has been reported that *SPOCK1* can regulate cell proliferation, migration, and apoptosis, and promote the development of several cancers (8, 9). Based on genome-wide association study (GWAS) correlation analyses, *SPOCK1* was identified as a new candidate gene in beef cattle (10) and human (11) menstrual cycle, suggesting that *SPOCK1* gene could be involved in animal reproductive life (12). It has been found that *SPOCK1* inhibits matrix metalloproteinase-2 (MMP-2) activation, which is a key factor in promoting endometrial menstrual rupture and the initiation of menstrual bleeding (13, 14). *SPOCK1* gene associated with female reproductive tract traits in polytocous sheep has the potential to regulate fertility and reproduction in the species (15). A disintegrin and metalloproteinase with thrombospondin-like repeats 1 (*ADAMTS1*) is located in granulosa cells (16) and plays an important role in the ovary through the progesterone receptor (PR)-dependent pathway (17). It is upregulated in preovulatory follicles of humans, mice, sheep, and cattle (18–20). Heparin-binding epidermal growth factor-like growth factor (*HBEGF*) inhibits apoptosis through the phosphatidylinositol-3 kinase/serine/threonine kinase (PI3K/Akt) signaling pathway (21), which is involved in early placental development and protects trophoblast cells from hypoxia-induced damage during pregnancy (22). Studies have also shown that *HBEGF* can be well-positioned to transmit paracrine near-secretory signals to blastocysts, which is related to the activation of uterine blastocysts during pregnancy (23, 24). *HBEGF* is a key regulator of embryo implantation in rodents, humans, and non-primates (22, 25). These results suggest that *SPOCK1, ADAMST1*, and *HBEGF* are closely related to the regulation of ovarian function, embryo implantation, and hormone synthesis, and have an effect on lambing numbers in sheep.

Leukemia inhibitory factor receptor (*LIFR*) belongs to the family of type I cytokine receptors and regulates cell proliferation and differentiation by activating the Janus Kinase/Signal Transducer and Activator of Transcription (JAK/STAT) signaling pathway. Mice deficient in *LIFR* show failure of implantation and metamorphosis, leading to embryonic death, suggesting that both the embryo and the uterus are potential targets for *LIFR* signaling (26, 27). Studies on the association between leukemia inhibitory factor (*LIF*) and lambing number in pigs have shown that LIFR plays an important role in embryo implantation (28). *LIF* is a key factor in regulating embryo implantation in many mammals (29). *LIFR* is expressed in the endometrium, preimplantation uterus, and placenta during embryo implantation, as well as in later stages of development (30). Although there have been limited studies on *LIFR* in sheep, it plays a crucial role in cell proliferation and embryonic development, affects reproduction, and can influence reproductive traits in sheep.

The above four candidate genes (*SPOCK1, ADAMTS1, HBEGF*, and *LIFR*) play a potential role in reproduction and so could be involved in sheep reproduction, resulting in differences in lambing number (15–18, 22, 29). SNPs in the above four genes were also found to be associated with reproductive traits in sheep in the pre-sequencing data. The obtained SNP annotation results and Fixation Index (Fst) were integrated and screened according to the following conditions: SNPs in introns with Fst > 0.15; non-synonymous mutation with Fst >0.05. In the present study, Sequenom MassARRAY^®^ SNP technology was used to detect the distribution of these four genes in five sheep breeds, categorized as polytocous and monotocous, and population genetics analysis was performed. Subsequently, bioinformatics analysis tools were used to predict the tertiary structure of candidate genes and their interacting proteins. The results provide new ideas and valuable genetic markers for sheep breeding.

## 2 Materials and methods

### 2.1 Animal preparation and sample collection

Ewes of five sheep breeds were included in this study, three of which were polytocous breeds, namely, small-tailed Han sheep (*n* = 384), Cele black sheep (*n* = 96), and Hu sheep (*n* = 96), and two were monotocous breeds, namely, Sunite sheep (*n* = 96), and Bamei mutton sheep (*n* = 96). The details of the five sheep breeds are displayed in Table 1. Blood samples were collected from all the sheep via the jugular vein, anticoagulated with EDTA, and stored at −20°C. The blood samples were collected at the same time from all the sheep that were uniformly bred, and details of the lambing numbers of small-tailed Han Sheep up to 3 years of age were recorded (refer to Supplementary Table S1). The other four breeds in this experiment were either all monotocous or polytocous breeds. However, the small-tailed Han sheep population is unique in that it encompasses individuals that produce both single and multiple lambs. Given this diversity, we have used the four breeds with established reproductive traits as reference points, and the small-tailed Han sheep with specific lambing records as our validation cohort. This strategy enables us to pinpoint potential genetic variations associated with lambing number, even when direct phenotypic data for all breeds are not available.

**Table 1:** Information on ewes used in this study.

**Breed**	**Group**	**Number**	**Sampling location**
Small-tailed Han sheep	Polytocous	384	Heze City, Shandong Province, China
Cele black sheep	Polytocous	96	Qira County, Hotan Prefecture, Xinjiang Uygur Autonomous Region, China
Hu sheep	Polytocous	96	Xuzhou City, Jiangsu Province, China
Sunite sheep	Monotocous	96	Urad Front Banner, Bayannaoer City, Inner Mongolia Autonomous Region, China
Bamei mutton sheep	Monotocous	96	Linhe District, Bayannaoer City, Inner Mongolia Autonomous Region, China

### 2.2 DNA extraction

DNA extraction from the sheep blood was performed using a DNA extraction kit (Tiangen Biochemical Technology Co., Ltd., Beijing, China). The steps are described in the instructions of the “Blood/Cell/Tissue Genomic DNA Extraction Kit” (catalog number: DP304). The concentration of the extracted DNA samples was determined using a Nanodrop 2000 (Thermo Fisher, USA), and the quality of the extracted DNA samples was evaluated by 1.5% agarose gel electrophoresis.

### 2.3 Genotyping

According to the sequence of sheep *SPOCK1, ADAMTS1, HBEGF*, and *LIFR* in GenBank ARS-UI_Ramb_v2.0 (accession number: XM_042250908.1, XM_004002797.5, XM_004003101.6, and XM_060400477.1), the single base extension primers of four loci (c.^*^1633 A>G, c.^*^1388T>C, and c.^*^1095G>A in SPOCK1, c.1847 A>G in *ADAMTS1*, c.^*^2403 A>G in *HBEGF*, and c.^*^127 T>C in *LIFR*) were designed by MassARRAY Assay Design v.3.1. The gene primers used in this study are shown in Table 2. These primers were synthesized by Beijing Compass Biotechnology Co., Ltd. (Beijing, China). Finally, Sequenom MassARRAY^®^ SNP technology was used to genotype the four loci of the polytocous groups (small-tailed Han sheep, Cele black sheep, and Hu sheep) and the monotocous groups (Sunite sheep and Bamei mutton sheep). For detailed information about the system and program, we referred to the previous research of our laboratory (31).

**Table 2:** Primer sequence information of the Sequenom MassARRAY^®^ SNP genotyping.

**Gene**	**Loci**	**Primer sequence (5^′^-3^′^)**
*SPOCK1*	c.^*^1633A>G	F-ACGTTGGATGTTGGGATAGGGATTGGTCAG
		R-ACGTTGGATGCATAAGGAGGCCACACAAAC
		EXT-CCACACAAACTCAGGCG
	c.^*^1388T>C	F-ACGTTGGATGGATTGTAGAGATGATGGCCC
		R-ACGTTGGATGACCGTTGCATTTCTTTGCCC
		EXT-CCCCTAAGTCAACTCCTGG
	c.^*^1095G>A	F-ACGTTGGATGATCCCCTGCTCTCTAAGAAG
		R-ACGTTGGATGTGATGTGTGGATAGATAGGG
		EXT-GGGAGTCTGCTAGGGAACAGCG
*ADAMTS1*	c.1847A>G	F-ACGTTGGATGACAGATCCTGCAACATCGAG
		R-ACGTTGGATGTCTTCAAGTGCCTGTTTGCG
		EXT-CTCTGCTCACCGTTATTC
*HBEGF*	c.^*^2403A>G	F-ACGTTGGATGGCAAGGCAAACAAGATCTGG
		R-ACGTTGGATGCTCCTTACTCCCATATAGCC
		EXT-GGCTACCATATAGCCCATTTCCA
*LIFR*	c.^*^127T>C	F-ACGTTGGATGGTTCTGTGGCTTTGAGACTG
		R-ACGTTGGATGTGTGAAGTGTTGCTAGTGGG
		EXT-CCGACTTCACTCTCACAAGTT

### 2.4 Polymerase chain reaction amplification and sanger sequencing

According to the results of genotyping and lambing number analysis of small-tailed Han sheep, the *LIFR* gene on chromosome 16 (Oar_v3.1) of sheep was selected for Polymerase Chain Reaction (PCR). Three DNA samples were randomly selected from each genotype for Sanger sequencing. Based on the annotation of *LIFR* SNP rs162302066 (Oar_v3.1) in the Ensembl gene annotation database, a 505-bp sequence was selected as the center to design primers for PCR amplification. The primer sequence is shown in Table 3. PCR amplification products were detected using agarose gel electrophoresis before Sanger sequencing. The primer amplification and Sanger sequencing were performed by Beijing Sangon Biotech Co., Ltd. The PCR reaction system included 8.91 μl of double-distilled water (ddH_2_O), 1 μl each of upstream and downstream primers (10 μM), 1.59 μl of template DNA ( ≤ 1 μg), and 12.5 μl of 2 × Master Mix. The amplification cycle included 30 cycles of pre-denaturation at 94°C for 3 min, followed by denaturation at 94°C for 30 s, annealing at 55°C for 30 s, extension at 72°C for 1 min, and final extension at 72°C for 5 min.

**Table 3:** Sequence information of PCR amplification primer for the *LIFR* gene.

**Gene**	**Primer sequence (5^′^-3^′^)**	**Length (bp)**	***T*_m_ (°C)**
*LIFR*	F-CACTGTAGACGACCTGGATGC	21	60
	R-ATTCTTTGGCTCGAATCCC	19	

### 2.5 Statistical analysis

The formulas for calculating allele and genotype frequencies, polymorphism information content (*PIC*), heterozygosity (*He*), and effective number of alleles (*Ne*) using genotyping data are as follows (32):


$$\begin{array}{l} PIC=1-\sum_{i=1}^{n}p_{i}^{2}-\sum_{i=1}^{n-1}\sum_{j=i+1}^{n}2p_{i}^{2}p_{j}^{2}\\ HE=1-\sum_{i=1}^{n}p_{i}^{2}\\ NE=1/\sum_{i=1}^{n}p_{i}^{2} \end{array}$$

where *n* is the number of alleles, *p*_*i*_ is the allele frequency of the *i*th allele, and *p*_*j*_ is the allele frequency of the *j*th allele.

The chi-squared test was used to detect whether the genotype distribution of each locus was in Hardy–Weinberg equilibrium. SPSS 26 (analysis of variance) was used to analyze the correlation between the four genotypes and lambing number. The following adjusted linear model was used:


$$\begin{array}{l} y=\text{μ }+P+G1+G2+G3+G4+e \end{array}$$

The least square mean of multiple comparisons of lambing number among different genotypes of small-tailed Han sheep was used. In the above equation, *y* is the phenotypic value (lambing number), μ is the population mean, *P* is the fixed parity effect, *G*1 is the fixed effect of candidate SNP in *SPOCK1*, *G*2 is the fixed effect of candidate SNP in *ADAMTS1*, *G*3 is the fixed effect of candidate SNP in *HBEGF*, *G*4 is the fixed effect of candidate SNP in *LIFR*, and *e* is the random error effect of each observation. At the same time, we also examined the interaction effect between each loci, in order to check whether the interaction between candidate loci needs to be supplemented in the model. However, no significant interaction was found between the candidate loci. Analysis was performed using R software (avo, Version 4.0.3).

### 2.6 Protein interaction network predicted by STRING database

For SNP loci significantly associated with lambing number, we predicted the protein interaction networks LIFR from the STRING database v.12.0 (https://cn.string-db.org) (33), which is a useful tool for predicting protein interactions.

## 3 Results

### 3.1 Genotyping and population genetic analysis of SNPs in candidate genes *SPOCK1, ADAMTS1, HBEGF*, and *LIFR*

**Figure 1:**
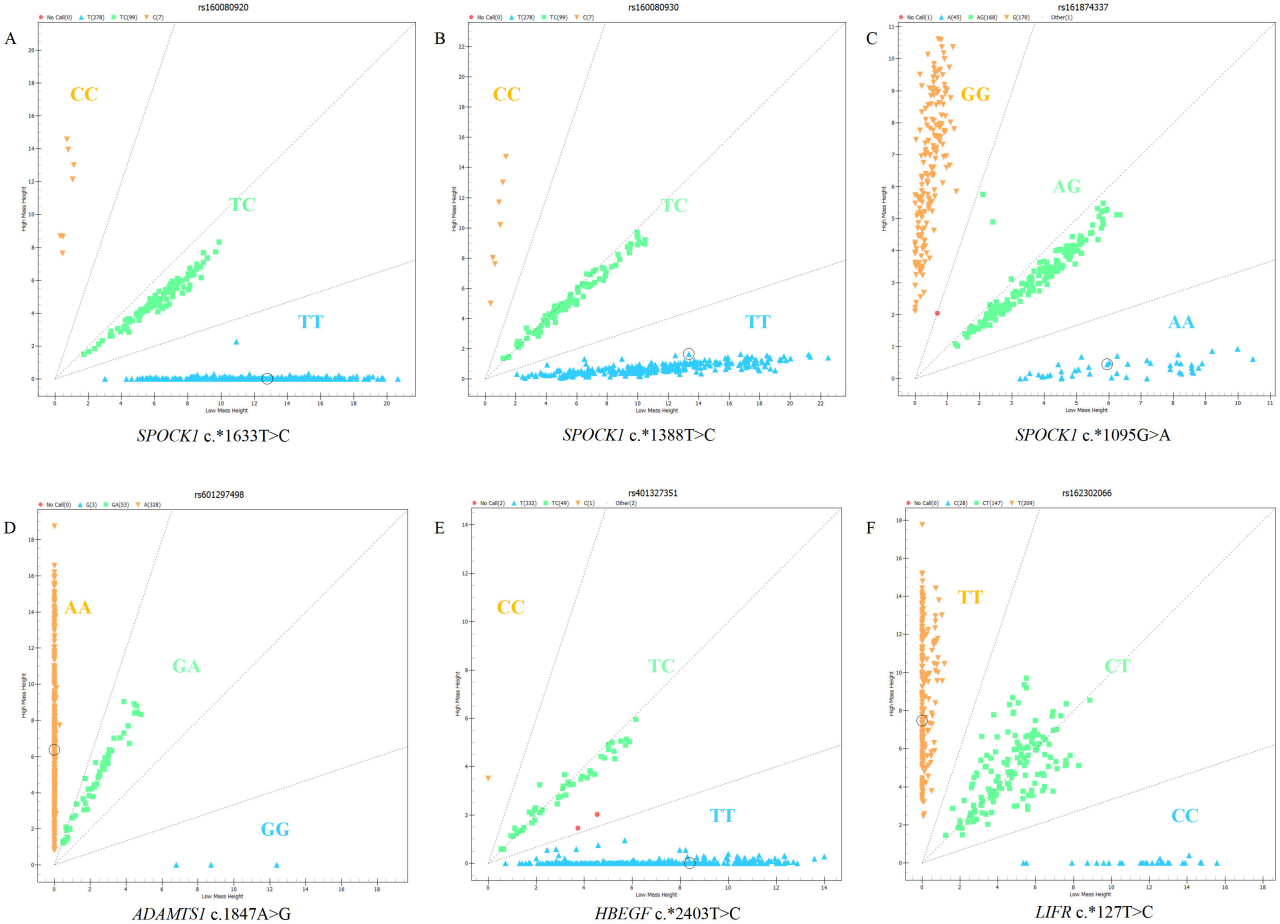
Genotyping results of candidate SNPs in *SPOCK1, ADAMTS1, HBEGF*, and *LIFR* genes using MassARRAY^®^ SNP system: **(A)** c.*1633T>C of *SPOCK1*, **(B)** c.*1388T>C of *SPOCK1*, **(C)** c.*1095G>A of *SPOCK1*, **(D)** c.1847A>G of *ADAMTS1*, **(E)** c.*2403T>C of *HBEGF*, and **(F)** c.*127T>C of *LIFR*.

The six SNPs (c.^*^1633 T>C, c.^*^1388T>C, and c.^*^1095G>A in SPOCK1, c.^*^1847 A>G in ADAMTS1, c.^*^2403 T>C in HBEGF, and c.^*^127 T>C in LIFR) were genotyped in five sheep breeds. The results showed that there were three genotypes in the SNP loci of the four genes, which could be visually distinguished by the MassARRAY^®^ SNP system (Figure 1).

The results of population genetic analysis of five sheep breeds (refer to Table 4) showed that the c.^*^1633 T>C and c.^*^1388T>C loci of SPOCK1 gene showed low polymorphism in small-tailed Han sheep and Bamei mutton sheep (PIC <0.25), and moderate polymorphism in Cele black sheep, Hu sheep, and Sunite sheep (0.25 ≤ PIC <0.5). The c.^*^1095G>A locus of the SPOCK1 gene showed low polymorphism in Bamei mutton sheep (PIC <0.25), and moderate polymorphism in small-tailed Han sheep, Cele black sheep, Hu sheep, and Sunite sheep (0.25 ≤ PIC <0.5). The c.1847A>G locus of the ADAMTS1 gene showed low polymorphism in five sheep breeds (PIC <0.25). The c.^*^2403 T>C locus of the HBEGF gene showed low polymorphism (PIC <0.25) in small-tailed Han sheep, Hu sheep, Sunite sheep, and Bamei mutton sheep, and moderate polymorphism (0.25 ≤ PIC <0.5) in Cele black sheep. The c.^*^127 T>C locus of the LIFR gene showed moderate polymorphism in five sheep breeds (0.25 ≤ PIC <0.5). According to the Hardy–Weinberg equilibrium, the c.^*^1633 T>C, c.^*^1388T>C, and c.^*^1095G>A loci of SPOCK1, the c.1847 A>G locus of ADAMTS1, and the c.^*^2403 T>C locus of HBEGF were in Hardy–Weinberg equilibrium in five sheep breeds (*p* > 0.05). The c.^*^127 T>C locus of the LIFR gene was in Hardy–Weinberg equilibrium in small-tailed Han sheep, Cele black sheep, Hu sheep, and Bamei mutton sheep (*p* > 0.05), and deviated from Hardy–Weinberg equilibrium in Sunite sheep (*p* < 0.05).

**Table 4:** Population polymorphism analysis of four candidate loci in five sheep breeds.

**Gene**	**SNPs**	**Breeds**	**Genotype frequency**			**Allele frequency**		**PIC**	***He***	***Ne***	**Chi-squared test (*p*-value)**
			**TT**	**TC**	**CC**	**T**	**C**				
*SPOCK1*	c.^*^1633T>C	Small-tailed Han sheep	0.72	0.26	0.02	0.85	0.15	0.22	0.25	1.34	0.59
		Cele black sheep	0.52	0.40	0.08	0.72	0.28	0.32	0.40	1.68	0.84
		Hu sheep	0.65	0.32	0.03	0.81	0.19	0.26	0.31	1.45	0.71
		Sunite sheep	0.63	0.34	0.03	0.80	0.20	0.27	0.32	1.48	0.54
		Bamei mutton sheep	0.73	0.23	0.04	0.84	0.16	0.23	0.26	1.36	0.20
			**TT**	**TC**	**CC**	**T**	**C**				
	c.^*^1388T>C	Small-tailed Han sheep	0.72	0.26	0.02	0.85	0.15	0.22	0.25	1.34	0.59
		Cele black sheep	0.53	0.39	0.08	0.72	0.28	0.32	0.40	1.67	0.73
		Hu sheep	0.66	0.31	0.03	0.81	0.19	0.26	0.31	1.44	0.80
		Sunite sheep	0.63	0.34	0.03	0.80	0.20	0.27	0.32	1.48	0.54
		Bamei mutton sheep	0.73	0.23	0.04	0.84	0.16	0.23	0.26	1.36	0.20
			**AA**	**AG**	**GG**	**A**	**G**				
	c.^*^1095G>A	Small-tailed Han sheep	0.12	0.44	0.44	0.34	0.66	0.35	0.45	1.81	0.72
		Cele black sheep	0.26	0.53	0.21	0.53	0.47	0.37	0.50	1.99	0.52
		Hu sheep	0.42	0.39	0.20	0.61	0.39	0.36	0.48	1.91	0.06
		Sunite sheep	0.08	0.51	0.41	0.34	0.66	0.35	0.45	1.81	0.17
		Bamei mutton sheep	0.05	0.24	0.71	0.17	0.83	0.24	0.28	1.40	0.12
			**AA**	**GA**	**GG**	**A**	**G**				
*ADAMTS1*	c.1847A>G	Small-tailed Han sheep	0.85	0.14	0.01	0.92	0.08	0.13	0.41	1.67	0.60
		Cele black sheep	0.96	0.04	0.00	0.98	0.02	0.04	0.04	1.04	0.83
		Hu sheep	0.95	0.05	0.00	0.97	0.03	0.05	0.05	1.05	0.79
		Sunite sheep	0.79	0.19	0.02	0.88	0.12	0.18	0.20	1.26	0.42
		Bamei mutton sheep	1.00	0.00	0.00	1.00	0.00	0.00	0.00	1.00	1.00
			**TT**	**TC**	**CC**	**T**	**C**				
*HBEGF*	c.^*^2403T>C	Small-tailed Han sheep	0.87	0.13	0.00	0.93	0.07	0.12	0.12	1.14	0.56
		Cele black sheep	0.54	0.41	0.05	0.74	0.26	0.31	0.38	1.61	0.50
		Hu sheep	0.93	0.07	0.00	0.96	0.04	0.07	0.07	1.08	0.71
		Sunite sheep	0.75	0.25	0.00	0.87	0.13	0.20	0.22	1.28	0.16
		Bamei mutton sheep	0.78	0.21	0.01	0.89	0.11	0.18	0.20	1.25	0.79
			**TT**	**CT**	**CC**	**T**	**C**				
*LIFR*	c.^*^127T>C	Small-tailed Han sheep	0.54	0.38	0.07	0.74	0.26	0.31	0.39	1.64	0.76
		Cele black sheep	0.60	0.35	0.05	0.78	0.22	0.28	0.34	1.52	0.72
		Hu sheep	0.40	0.40	0.21	0.59	0.41	0.37	0.48	1.93	0.08
		Sunite sheep	0.55	0.30	0.15	0.70	0.30	0.33	0.42	1.72	0.01
		Bamei mutton sheep	0.07	0.48	0.45	0.31	0.69	0.34	0.43	1.75	0.26

Ne, number of effective alleles; He, heterozygosity; SNPs, single nucleotide polymorphisms; PIC, polymorphism information content.

PIC > 0.5 is high polymorphism, 0.25 ≤ PIC ≤ 0.5 is moderate polymorphism, PIC <0.25 is low polymorphism; *p* > 0.05 is in Hardy–Weinberg equilibrium; *p* < 0.05 is not in Hardy–Weinberg equilibrium.

### 3.2 Genotyping and allele frequencies of SNPs in the four genes of polytocous and monotocous sheep

According to the lambing number, the five breeds were divided into two categories: polytocous sheep (small-tailed Han sheep, Cele black sheep, and Hu sheep) and monotocous sheep (Sunite sheep and Bamei mutton sheep). Genotyping of six SNPs in the five sheep breeds is shown in Table 5. There was no significant difference in genotype frequency and allele frequency of c.^*^1633 T>C locus and c.^*^1388T>C locus of the *SPOCK1* gene, c.1847 A>G locus of the *ADAMTS1* gene, and c.^*^2403 T>C locus of the *HBEGF* gene between polytocous and monotocous sheep breeds (*p* > *0.05*). The genotype and allele frequencies of c.^*^1095G>A locus of the *SPOCK1* gene and c.^*^127 T>C locus of the *LIFR* gene were significantly different between polytocous and monotocous sheep breeds (*p* < 0.05).

**Table 5:** Genotype and allele frequencies of SNP loci in populations of sheep with different reproductive capacities.

**Gene**	**SNPs**	**Types of fecundity**	**Genotype frequency (count)**			**Chi-squared test (*p-*value)**	**Allele frequency**		**Chi-squared test (*p-*value)**
			**TT**	**TC**	**CC**		**T**	**C**	
*SPOCK1*	c.^*^1633 T>C	Polytocous sheep	0.68 (390)	0.29 (168)	0.03 (18)	0.94	0.82	0.18	0.91
		Monotocous sheep	0.68 (130)	0.28 (55)	0.04 (7)	0.95	0.82	0.18	0.92
			**TT**	**TC**	**CC**		**T**	**C**	
	c.^*^1388T>C	Polytocous sheep	0.68 (392)	0.29 (166)	0.03 (18)	0.94	0.82	0.18	0.85
		Monotocous sheep	0.68 (130)	0.29 (55)	0.03 (192)	0.95	0.82	0.81	0.87
			**AA**	**AG**	**GG**		**A**	**G**	
	c.^*^1095G>A	Polytocous sheep	0.19 (110)	0.45 (256)	0.36 (209)	0.00	0.41	0.59	0.00
		Monotocous sheep	0.07 (13)	0.37 (72)	0.56 (109)	0.00	0.26	0.74	0.00
			**AA**	**GA**	**GG**		**A**	**G**	
*ADAMTS1*	c.1847 A>G	Polytocous sheep	0.88 (508)	0.11 (62)	0.01 (3)	0.59	0.94	0.06	0.84
		Monotocous sheep	0.90 (167)	0.09 (17)	0.01 (2)	0.67	0.94	0.06	0.86
			**TT**	**TC**	**CC**		**T**	**C**	
*HBEGF*	c.^*^2403 T>C	Polytocous sheep	0.82 (473)	0.17 (95)	0.01 (6)	0.11	0.91	0.09	0.12
		Monotocous sheep	0.76 (146)	0.23 (44)	0.01 (1)	0.19	0.88	0.12	0.18
			**TT**	**CT**	**CC**		**T**	**C**	
*LIFR*	c.^*^127 T>C	Polytocous sheep	0.53 (305)	0.38 (219)	0.09 (52)	0.00	0.72	0.28	0.00
		Monotocous sheep	0.31 (59)	0.39 (75)	0.30 (57)	0.00	0.51	0.49	0.00

### 3.3 Association analysis of SNPs in SPOCK1, ADAMTS1, HBEGF, and LIFR with lambing number in small-tailed Han sheep

The association analysis of SNPs in *SPOCK1, ADAMTS1, HBEGF*, and *LIFR* genes with lambing number in Small-tailed Han sheep is shown in Table 6. There was a significant association between the c.^*^127 T>C locus of *LIFR* and the second, third, and average lambing number of small-tailed Han sheep (*p* < 0.05). The lambing number of ewes with the CC genotype was significantly lower than that with the TT and CT genotypes (*p* < 0.05). The results showed that the mutation site of *LIFR* c.^*^127 T>C could be involved in the regulation of lambing number in small-tailed Han sheep to a certain extent, while the other loci were not significantly associated with the lambing number of small-tailed Han sheep (*p* > 0.05).

**Table 6:** Least squares mean and standard errors of the lambing number in small-tailed Han sheep with different genotypes.

**Gene**	**SNPs**	**Genotypes (number)**	**1st parity lambing number**	**2nd parity lambing number**	**3rd parity lambing number**	**Average lambing number**
*SPOCK1*	c.^*^1633T>C	TT (278)	2.00 ± 0.057	1.69 ± 0.081	0.75 ± 0.083	1.48 ± 0.054
		TC (99)	1.99 ± 0.119	1.76 ± 0.135	0.68 ± 0.135	1.47 ± 0.099
		CC (7)	1.86 ± 0.508	2.29 ± 0.565	1.29 ± 0.644	1.81 ± 0.508
	c.^*^1388T>C	TT (278)	2.00 ± 0.057	1.69 ± 0.081	0.75 ± 0.083	1.53 ± 0.055
		TC (99)	1.99 ± 0.119	1.76 ± 0.135	0.68 ± 0.135	1.46 ± 0.101
		CC (7)	1.86 ± 0.508	2.29 ± 0.565	1.29 ± 0.644	1.71 ± 0.565
	c.^*^1095G>A	AA (45)	1.76 ± 0.159	1.93 ± 0.214	0.80 ± 0.207	1.47 ± 0.158
		AG (167)	2.07 ± 0.081	1.77 ± 0.103	0.81 ± 0.115	1.55 ± 0.078
		GG (171)	1.99 ± 0.074	1.63 ± 0.104	0.67 ± 0.097	1.50 ± 0.066
*ADAMTS1*	c.1847A>G	AA (328)	1.98 ± 0.056	1.74 ± 0.074	0.75 ± 0.076	1.49 ± 0.051
		GA (53)	2.09 ± 0.143	1.58 ± 0.195	0.72 ± 0.189	1.47 ± 0.138
		GG (3)	2.00 ± 0.577	2.00 ± 1.155	—	1.33 ± 0.509
*HBEGF*	c.^*^2403T>C	TT (332)	1.97 ± 0.056	1.67 ± 0.074	0.73 ± 0.075	1.46 ± 0.051
		TC (49)	2.18 ± 0.142	2.06 ± 0.192	0.80 ± 0.204	1.68 ± 0.130
		CC (1)	1.00 ± 0.000	3.00 ± 0.000	—	1.33 ± 0.000
*LIFR*	c.^*^127T>C	TT (208)	2.03 ± 0.070	1.76 ± 0.091^a^	0.85 ± 0.103^a^	1.55 ± 0.066^a^
		CT (148)	1.98 ± 0.085	1.78 ± 0.116^a^	0.70 ± 0.107^a^	1.48 ± 0.077^a^
		CC (28)	1.79 ± 0.188	1.07 ± 0.230^b^	0.14 ± 0.991^b^	1.00 ± 0.106^b^

The number in brackets denotes the number of ewes with the kind of genotype. Different lower-case letters next to the mean lambing number indicate that there were significant differences between genotypes (*p* < 0.05).

### 3.4 SNP site sequencing of the *LIFR* candidate gene

**Figure 2:**
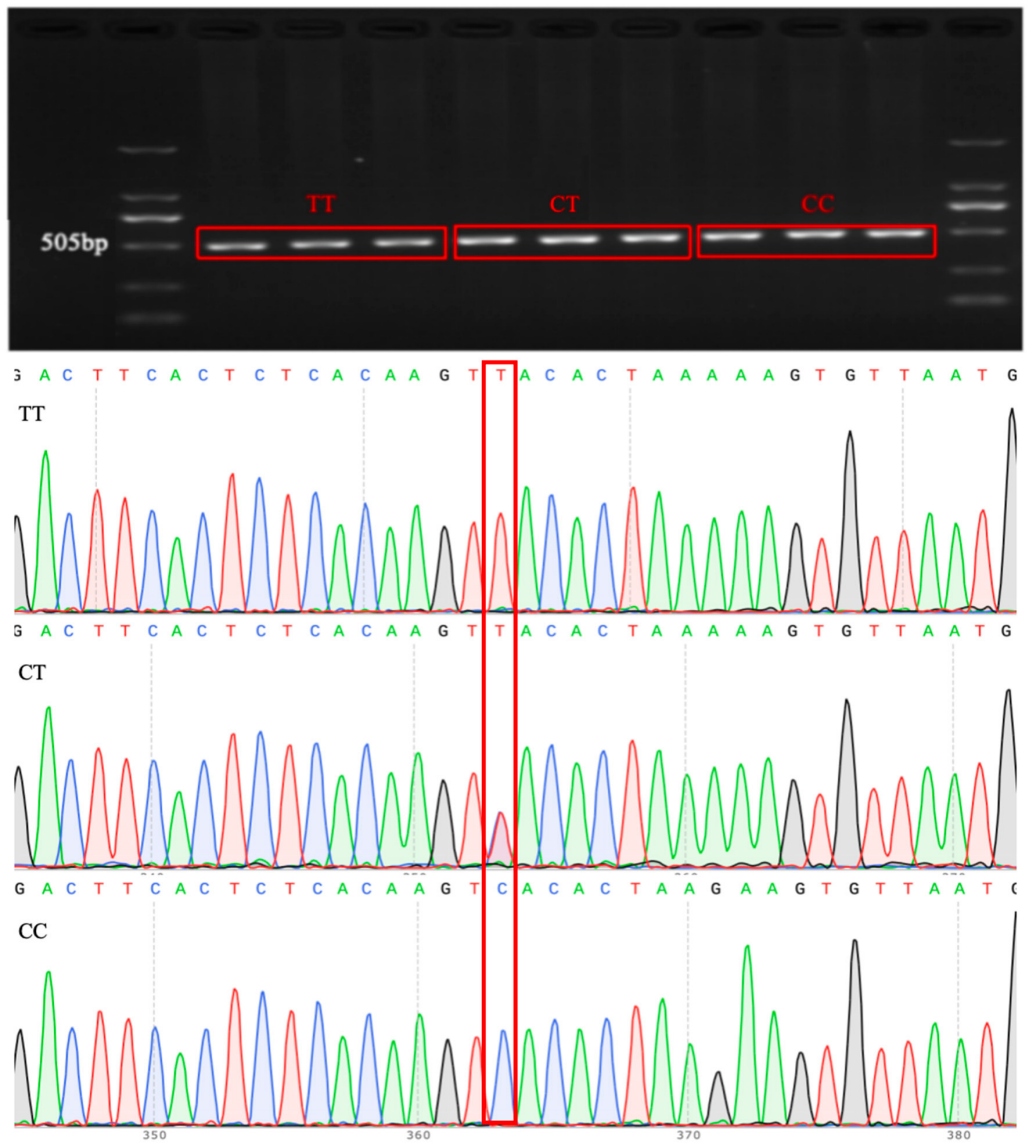
Electrophoresis results of PCR products and sequencing results for the *LIFR* SNP c.*127T>C locus.

Association analysis between candidate genes and the lambing number of small-tailed Han sheep showed that there was a significant difference between the *LIFR* candidate gene and lambing number in this breed. Genotyping of *LIFR* SNP c.^*^127T>C revealed the presence of three genotypes of this locus in the small-tailed Han sheep population, namely, TT (209, 54.4%), CT (147, 38.3%), and CC (28, 7.3%), with a detection rate of >99% in 384 samples (Figure 1). The results of PCR amplification and Sanger sequencing further illustrated the existence of *LIFR* SNP c.^*^127T>C and the reliability of the typing results (Figure 2).

### 3.5 Structure and interaction network analysis of the LIFR protein

**Figure 3:**
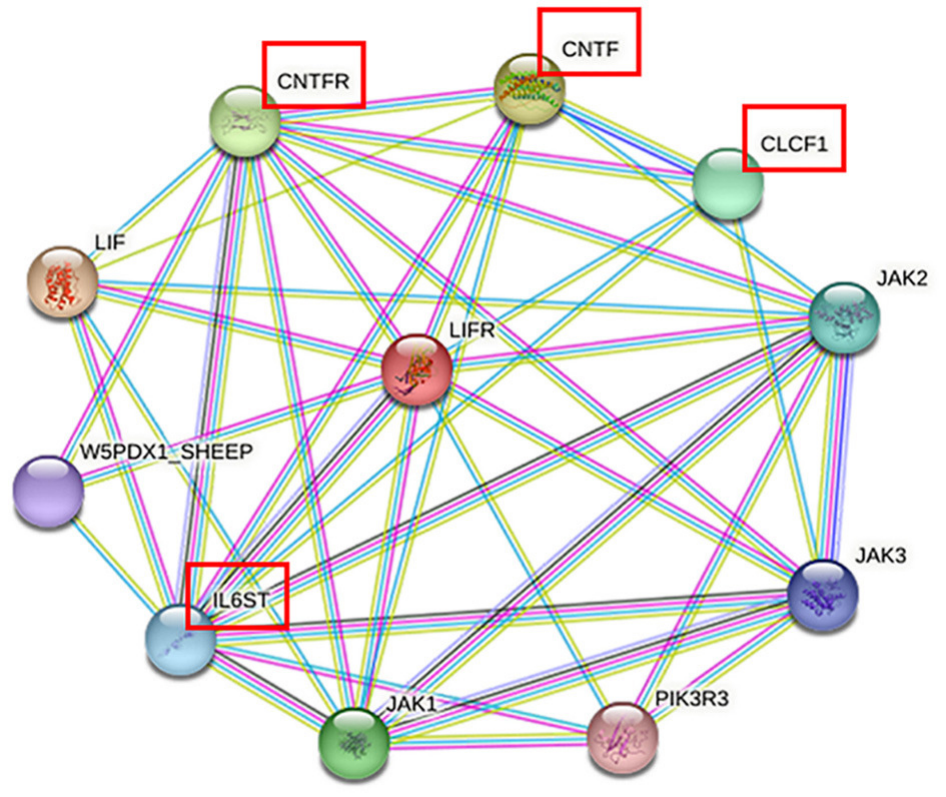
Protein networks closely related to LIFR in sheep.

To further explore the biological function of the *LIFR* gene in reproductive traits in sheep, a network of closely related proteins was constructed using the STRING website. Among the 10 proteins that interacted most strongly with LIFR (Figure 3), the key proteins that were closely related to lambing number, such as ciliary neurotrophic factor (CNTF), cardiotrophin-like cytokine factor 1 (CLCF1), interleukin 6 cytokine family signal transducer (IL6ST), and ciliary neurotrophic factor receptor (CNTFR), were identified based on interacting protein information (refer to Table 7) and were found to be closely related to reproduction, such as development and embryo implantation (34–36). These findings further suggest that LIFR is an important factor influencing lamb numbers in sheep.

**Table 7:** Information on the 10 proteins that interact most strongly with LIFR.

**Description**	**Abbreviation expansion**	**Function**	**Score**
CNTFR	Ciliary neurotrophic factor receptor	Embryonic development and nervous system	0.967
CNTF	Ciliary neurotrophic factor	Embryo implantation	0.988
CLCF1	Cardiotrophin-like cytokine factor 1	Embryonic development and muscle function	0.960
JAK2	Janus kinase 2	Immune response, hematopoiesis, and metabolic regulation	0.960
JAK3	Janus kinase 3	Immune response, hematopoiesis and metabolic regulation	0.934
PIK3R3	Phosphoinositide-3-kinase regulatory subunit 3	Regulates cell growth, survival, and metabolism	0.914
JAK1	Janus kinase 1	Immune response, hematopoiesis, and metabolic regulation	0.961
IL6ST	Interleukin 6 cytokine family signal transducer	Embryonic development, nervous system and muscle function	0.952
W5PDX1_SHEEP	Uncharacterized protein	Function not yet clear	0.917
LIF	Leukemia inhibitory factor		0.988

## 4 Discussion

We previously screened four candidate genes (*SPOCK1, ADAMTS1, HBEGF*, and *LIFR*) associated with lambing number in sheep by GWAS analysis and selective signal scanning (15, 22, 37). Their association with lambing number in sheep needs to be further clarified. In this study, the four candidate genes mentioned above were validated by amplification using Sequenom MassARRAY^®^ SNP technology.

In this study, specifically, mutation loci including c.^*^1633T>C, c.^*^1388T>C, and c.^*^1095G>A for *SPOCK1*, c.1847A>G for *ADAMTS1*, and c.^*^2403T>C for *HBEGF* exhibited low polymorphism and moderate polymorphism. This suggests that the level of genetic variation of these loci varies in different breeds, which may have an impact on the genetic diversity and adaptability of the breed. On the other hand, the c.^*^127T>C mutation of *LIFR* showed moderate polymorphism, suggesting that the level of genetic variation at this locus is relatively high. This polymorphism may contribute to the phenotypic diversity observed in sheep populations. Meanwhile, analyses of Hardy–Weinberg equilibrium revealed interesting patterns. The c.^*^127T>C mutation in *LIFR* was found to significantly deviate from Hardy–Weinberg equilibrium (*p* < 0.05) in a population of Sunite sheep, suggesting that certain evolutionary forces, such as selection, mutation, migration, or non-random mating, may act on these loci. In contrast, c.^*^1633T>C, c.^*^1388T>C, and c.^*^1095G>A of *SPOCK1*, c.1847A>G of *ADAMTS1*, and c.^*^2403T>C of *HBEGF* were all found to be in Hardy–Weinberg equilibrium. Additionally, the c.^*^127T>C mutation of LIFR in the other four sheep breeds was also in Hardy–Weinberg equilibrium. This suggests that these loci are relatively stable under current population conditions and are not subject to strong evolutionary pressures. In addition, we found that the genotype frequencies and allele frequencies of the c.^*^1095G>A locus of the *SPOCK1* gene and the c.^*^127 T>C locus of the *LIFR* gene differed significantly (*p* < 0.05) between polytocous and monotocous sheep. This implies that these loci are significantly associated with lambing traits. In contrast, the genotype frequencies and allele frequencies of the c.^*^1633 T>C locus and c.^*^1388 T>C locus of the *SPOCK1* gene, the c.1847 A>G locus of the *ADAMTS1* gene, and the c.^*^2403 T>C locus of the *HBEGF* gene were not significantly different between multiparous and singleton sheep (*p* > 0.05), which may indicate that these loci are not significantly associated with lambing traits in sheep. In addition to genetic analyses of the identified SNPs, a review of the existing literature on *LIFR* function provided valuable insights.

In the present study, we also carried out an association analysis of the SNP loci of *SPOCK1, ADAMTS1, HBEGF*, and *LIFR* genes with the lambing number of small-tailed Han sheep and found that the c.^*^127 T>C locus of the *LIFR* gene was also significantly correlated with the second and third lambing numbers and the average lambing number of the small-tailed Han sheep. The number of lambs in ewes with TT and CT genotypes was significantly higher than that in ewes with the CC genotype. This is in agreement with previous findings (28), which suggests that *LIFR* may be a potential genetic marker influencing the number of lambs in sheep reproduction. SNPs in the *LIFR* gene were successfully identified by Sanger sequencing. The presence of *LIFR* SNPs in the small-tailed Han sheep population was established, indicating the presence of the *LIFR* SNP c.^*^127 T>C and the reliability of the typing results. In addition, as a transmembrane receptor of leukemia inhibitory factor (*LIF*), *LIFR* is widely considered to be a key factor in regulating mammalian embryo implantation (29). Moreover, *LIF* can inhibit the differentiation of mouse embryonic stem cells (ES), suggesting that this cytokine regulates uterine function and embryonic development. Histologically, the lack of well-organized glial cells and labyrinthine layers in the placenta suggests that embryonic lethality is due to changes in trophoblast cell differentiation and that *LIFR* signaling plays an important role in promoting embryonic trophoblast cell differentiation (38, 39). Moreover, *LIFR* transmits *LIF* signals through downstream channels (40). Disruption of *LIFR* results in decreased embryo survival. *LIFR* may be a ligand for the activation of Signal Transducer and Activator of Transcription 3 (STAT3), which has been shown to play a role in mammary degenerative apoptosis (41). LIF/STAT3 signaling is consistent with the induction of apoptosis in mammary epithelial cells (42), suggesting that increased expression of *LIFR* in granulosa cells leads to increased STAT3 phosphorylation. Normal embryo implantation and development, as well as follicular atresia, have an important impact on mammalian fertility and are also key factors affecting the number of lambs produced by the small-tailed Han sheep. This further suggests that LIFR is involved in the reproductive process of sheep and can influence the number of lambs produced by sheep by affecting embryo development, which is consistent with the result of this study that *LIFR* can affect reproductive traits in sheep.

The majority of physiological processes are influenced by multiple proteins. Therefore, to better understand the role of *LIFR* genes in reproductive traits in sheep, a protein–protein interaction network was established to deepen our understanding of complex traits such as reproduction. Based on the protein–protein interaction network provided by the STRING database, we predicted 10 proteins that interact closely with LIFR. The proteins Janus kinase (JAK1), Janus kinase 2 (JAK2), and Janus kinase 3 (JAK3) are important members of the JAK family, which are involved in the signaling of a variety of cytokines and growth factors, and are essential for immune response, hematopoiesis, and metabolic regulation. Phosphoinositide-3-kinase regulatory subunit 3 (PIK3R3) is a key regulator of the PI3K signaling pathway, affecting cell growth and survival. However, four proteins, CNTF, CLCF1, IL6ST, and CNTFR, were related to reproduction, such as development and embryo implantation (35, 43, 44). Furthermore, all belong to the interleukin 6 (IL6) family (45, 46). Its stimulation by interferon tau (IFNT) and regulation by Progesterone 4 (P4) in a complex physiological phase and cell-specific manner is associated with the acceptability of intrauterine implantation in many species, including humans, rodents, pigs, cattle, mink and sheep (45, 47). CNTF plays an important role in the production of luteinizing hormone (LH) and prolactin (PRL) before ovulation in rats. The absence of CNTF will affect the production of LH, which in turn affects the ovulation rate (48). IL6 mediates early embryo implantation and placenta development through ovarian steroid hormones, estrogen, and progesterone (49). It indicates that CNTF, CLCF1, IL6ST, and CNTFR are involved in the regulation of reproductive processes. In conclusion, LIFR genes are associated with the regulation of lambing numbers in sheep. Thus, these networks further confirm that the c.^*^127 T>C locus of the *LIFR* gene can be used as a potential molecular marker for the lambing number trait in small-tailed Han sheep. However, the interactions between these genes and how they synergistically regulate embryo implantation, follicular development, and ovulation remain to be further explored. It was also found that *LIFR* can influence lambing number in sheep through its function in embryonic development. the significant correlation between the c.^*^127 T>C locus of the *LIFR* gene and lambing number suggests that this SNP could serve as a valuable molecular marker in sheep breeding programs, thereby improving the economics of sheep production.

## 5 Conclusion

In this study, the association between SNPs of *SPOCK1, ADAMTS1, HBEGF*, and *LIFR* and lambing number in small-tailed Han sheep was analyzed. The results showed that the genotype frequency and allele frequency of the *LIFR* gene c.^*^127 T>C locus were significantly different between polytocous and monotocous sheep breeds. There was a significant association between the c.^*^127T>C locus of the *LIFR* gene and lambing number in small-tailed Han sheep. It is speculated that there may be a correlation between this locus and the lambing number trait in sheep. Therefore, this gene locus can be used as a potential genetic marker for sheep breeding.

## Data Availability

The original contributions presented in the study are included in the article/Supplementary material, further inquiries can be directed to the corresponding authors.
