# Supplementary material for: Association analysis of single nucleotide polymorphisms in the LIFR gene with lambing number in sheep
Source: Front Vet Sci. 2025 Jul 10;12:1629162. doi: 10.3389/fvets.2025.1629162 (PMC12287739; doi:10.3389/fvets.2025.1629162)
Supplement: Supplementary file 1 [file Table_1.docx]

Supplementary Table 1. Lambing number statistics for Small-tailed Han Sheep

| number | genotype | 1^st^ parity lambing number | 2^nd^ parity lambing number | 3^rd^ parity lambing number | number | genotype | 1^st^ parity lambing number | 2^nd^ parity lambing number | 3^rd^ parity lambing number |
| --- | --- | --- | --- | --- | --- | --- | --- | --- | --- |
| 1 | TT | 2 | 2 | 3 | 193 | T | 2 | 2 | 0 |
| 2 | CT | 3 | 0 | 0 | 194 | T | 1 | 2 | 0 |
| 3 | TT | 3 | 3 | 0 | 195 | T | 3 | 4 | 5 |
| 4 | TT | 4 | 2 | 3 | 196 | T | 3 | 3 | 3 |
| 5 | TT | 2 | 1 | 0 | 197 | T | 3 | 4 | 3 |
| 6 | CT | 1 | 3 | 0 | 198 | T | 2 | 3 | 0 |
| 7 | TT | 1 | 2 | 0 | 199 | T | 3 | 1 | 3 |
| 8 | CC | 2 | 0 | 0 | 200 | CT | 3 | 3 | 0 |
| 9 | TT | 3 | 3 | 3 | 201 | T | 3 | 3 | 4 |
| 10 | TT | 3 | 3 | 0 | 202 | CT | 2 | 2 | 0 |
| 11 | TT | 2 | 5 | 5 | 203 | CT | 2 | 3 | 0 |
| 12 | TT | 2 | 3 | 4 | 204 | T | 2 | 3 | 4 |
| 13 | TT | 2 | 2 | 2 | 205 | T | 1 | 2 | 1 |
| 14 | CT | 2 | 3 | 3 | 206 | T | 2 | 2 | 3 |
| 15 | CT | 3 | 0 | 0 | 207 | T | 4 | 4 | 5 |
| 16 | CT | 3 | 4 | 0 | 208 | T | 3 | 3 | 0 |
| 17 | TT | 1 | 2 | 0 | 209 | T | 3 | 4 | 3 |
| 18 | TT | 2 | 2 | 2 | 210 | CT | 3 | 0 | 0 |
| 19 | TT | 2 | 3 | 3 | 211 | T | 2 | 3 | 0 |
| 20 | TT | 1 | 2 | 2 | 212 | T | 2 | 3 | 2 |
| 21 | TT | 1 | 0 | 0 | 213 | T | 3 | 2 | 3 |
| 22 | TT | 1 | 1 | 0 | 214 | T | 3 | 3 | 4 |
| 23 | TT | 2 | 3 | 5 | 215 | T | 2 | 1 | 2 |
| 24 | T | 2 | 2 | 3 | 216 | CT | 0 | 2 | 3 |
| 25 | T | 2 | 3 | 2 | 217 | CT | 4 | 3 | 0 |
| 26 | CT | 2 | 0 | 0 | 218 | T | 2 | 1 | 5 |
| 27 | T | 2 | 3 | 0 | 219 | T | 2 | 2 | 0 |
| 28 | T | 2 | 2 | 0 | 220 | CT | 2 | 3 | 0 |
| 29 | T | 2 | 3 | 2 | 221 | T | 2 | 3 | 0 |
| 30 | T | 2 | 3 | 3 | 222 | CT | 2 | 1 | 2 |
| 31 | CT | 3 | 0 | 0 | 223 | T | 2 | 2 | 3 |
| 32 | T | 3 | 0 | 0 | 224 | T | 2 | 0 | 0 |
| 33 | T | 2 | 0 | 0 | 225 | T | 2 | 2 | 0 |
| 34 | T | 2 | 2 | 3 | 226 | CT | 2 | 3 | 2 |
| 35 | CT | 1 | 0 | 0 | 227 | CT | 2 | 2 | 0 |
| 36 | T | 3 | 0 | 0 | 228 | T | 1 | 0 | 0 |
| 37 | T | 3 | 3 | 0 | 229 | T | 2 | 2 | 0 |
| 38 | CT | 0 | 1 | 0 | 230 | T | 2 | 3 | 0 |
| 39 | CT | 0 | 2 | 0 | 231 | CT | 1 | 0 | 0 |
| 40 | C | 0 | 2 | 0 | 232 | T | 2 | 2 | 0 |
| 41 | T | 2 | 0 | 0 | 233 | T | 3 | 2 | 0 |
| 42 | T | 2 | 0 | 0 | 234 | CT | 1 | 2 | 3 |
| 43 | CT | 1 | 0 | 0 | 235 | CT | 3 | 3 | 3 |
| 44 | CT | 0 | 2 | 0 | 236 | CT | 2 | 0 | 0 |
| 45 | CT | 1 | 0 | 0 | 237 | T | 1 | 0 | 0 |
| 46 | T | 0 | 2 | 0 | 238 | T | 2 | 3 | 0 |
| 47 | CT | 0 | 2 | 0 | 239 | T | 3 | 2 | 0 |
| 48 | C | 1 | 0 | 0 | 240 | T | 1 | 0 | 0 |
| 49 | T | 0 | 1 | 0 | 241 | T | 2 | 2 | 2 |
| 50 | T | 0 | 1 | 0 | 242 | CT | 2 | 3 | 0 |
| 51 | CT | 2 | 0 | 0 | 243 | T | 3 | 2 | 0 |
| 52 | CT | 2 | 0 | 0 | 244 | C | 1 | 2 | 2 |
| 53 | CT | 0 | 1 | 0 | 245 | T | 3 | 2 | 0 |
| 54 | T | 2 | 0 | 0 | 246 | T | 2 | 3 | 0 |
| 55 | T | 3 | 0 | 0 | 247 | CT | 1 | 0 | 0 |
| 56 | T | 0 | 2 | 0 | 248 | CT | 2 | 0 | 0 |
| 57 | CT | 0 | 1 | 0 | 249 | CT | 1 | 3 | 0 |
| 58 | T | 0 | 1 | 0 | 250 | T | 2 | 3 | 0 |
| 59 | T | 0 | 1 | 0 | 251 | T | 1 | 3 | 0 |
| 60 | T | 0 | 2 | 0 | 252 | CT | 2 | 0 | 0 |
| 61 | T | 0 | 2 | 0 | 253 | T | 2 | 0 | 0 |
| 62 | T | 0 | 1 | 0 | 254 | T | 3 | 0 | 0 |
| 63 | T | 0 | 1 | 0 | 255 | T | 2 | 0 | 0 |
| 64 | CT | 0 | 3 | 0 | 256 | T | 2 | 0 | 0 |
| 65 | T | 0 | 1 | 0 | 257 | T | 2 | 3 | 6 |
| 66 | T | 1 | 0 | 0 | 258 | CT | 3 | 4 | 3 |
| 67 | C | 0 | 1 | 0 | 259 | CT | 3 | 4 | 0 |
| 68 | T | 2 | 0 | 0 | 260 | CT | 2 | 5 | 3 |
| 69 | T | 2 | 0 | 0 | 261 | T | 2 | 1 | 0 |
| 70 | CT | 0 | 2 | 0 | 262 | T | 4 | 3 | 3 |
| 71 | T | 1 | 0 | 0 | 263 | T | 3 | 3 | 0 |
| 72 | T | 0 | 2 | 0 | 264 | T | 3 | 4 | 5 |
| 73 | T | 0 | 2 | 0 | 265 | CT | 3 | 4 | 3 |
| 74 | CT | 0 | 2 | 0 | 266 | CT | 2 | 2 | 0 |
| 75 | CT | 2 | 0 | 0 | 267 | C | 2 | 2 | 0 |
| 76 | CT | 2 | 0 | 0 | 268 | T | 3 | 2 | 0 |
| 77 | T | 0 | 3 | 0 | 269 | CT | 2 | 3 | 0 |
| 78 | CT | 0 | 2 | 0 | 270 | C | 2 | 0 | 0 |
| 79 | T | 1 | 0 | 0 | 271 | CT | 2 | 3 | 3 |
| 80 | T | 0 | 2 | 0 | 272 | T | 2 | 1 | 1 |
| 81 | CT | 0 | 2 | 0 | 273 | T | 2 | 2 | 3 |
| 82 | CT | 2 | 0 | 0 | 274 | T | 2 | 2 | 0 |
| 83 | T | 0 | 2 | 0 | 275 | CT | 2 | 0 | 0 |
| 84 | T | 0 | 2 | 0 | 276 | CT | 2 | 2 | 0 |
| 85 | C | 0 | 2 | 0 | 277 | CT | 1 | 0 | 0 |
| 86 | T | 2 | 0 | 0 | 278 | T | 1 | 2 | 0 |
| 87 | C | 2 | 0 | 0 | 279 | T | 2 | 4 | 0 |
| 88 | CT | 2 | 0 | 0 | 280 | T | 2 | 2 | 2 |
| 89 | CT | 1 | 0 | 0 | 281 | T | 5 | 3 | 0 |
| 90 | T | 0 | 2 | 0 | 282 | CT | 4 | 3 | 4 |
| 91 | C | 2 | 0 | 0 | 283 | CT | 2 | 4 | 0 |
| 92 | CT | 2 | 0 | 0 | 284 | T | 3 | 2 | 0 |
| 93 | T | 0 | 2 | 0 | 285 | T | 3 | 4 | 0 |
| 94 | T | 0 | 2 | 0 | 286 | T | 2 | 1 | 1 |
| 95 | CT | 0 | 2 | 0 | 287 | T | 3 | 4 | 0 |
| 96 | CT | 0 | 3 | 0 | 288 | T | 2 | 0 | 0 |
| 97 | T | 3 | 3 | 4 | 289 | T | 3 | 4 | 0 |
| 98 | C | 1 | 0 | 0 | 290 | T | 3 | 0 | 0 |
| 99 | CT | 2 | 0 | 3 | 291 | CT | 3 | 2 | 0 |
| 100 | CT | 1 | 0 | 0 | 292 | CT | 4 | 0 | 0 |
| 101 | T | 1 | 0 | 3 | 293 | T | 4 | 5 | 5 |
| 102 | CT | 2 | 1 | 0 | 294 | T | 3 | 4 | 4 |
| 103 | CT | 1 | 0 | 0 | 295 | T | 3 | 0 | 0 |
| 104 | CT | 2 | 0 | 0 | 296 | T | 3 | 0 | 0 |
| 105 | T | 2 | 0 | 0 | 297 | T | 2 | 2 | 0 |
| 106 | T | 2 | 0 | 0 | 298 | CT | 1 | 0 | 0 |
| 107 | CT | 2 | 4 | 0 | 299 | T | 2 | 0 | 0 |
| 108 | CT | 2 | 2 | 0 | 300 | CT | 2 | 2 | 0 |
| 109 | T | 2 | 0 | 0 | 301 | C | 3 | 3 | 0 |
| 110 | T | 2 | 0 | 0 | 302 | CT | 2 | 0 | 0 |
| 111 | CT | 2 | 0 | 0 | 303 | T | 3 | 0 | 0 |
| 112 | T | 2 | 0 | 0 | 304 | T | 2 | 2 | 4 |
| 113 | T | 2 | 0 | 0 | 305 | T | 3 | 2 | 0 |
| 114 | T | 2 | 0 | 0 | 306 | CT | 2 | 2 | 0 |
| 115 | CT | 2 | 2 | 2 | 307 | CT | 2 | 3 | 3 |
| 116 | T | 2 | 0 | 0 | 308 | CT | 3 | 2 |  |
| 117 | T | 2 | 0 | 0 | 309 | CT | 2 | 4 | 2 |
| 118 | C | 2 | 0 | 0 | 310 | T | 2 | 2 | 2 |
| 119 | C | 4 | 3 | 0 | 311 | CT | 4 | 4 | 4 |
| 120 | T | 3 | 0 | 0 | 312 | T | 4 | 4 | 4 |
| 121 | T | 4 | 0 | 0 | 313 | CT | 4 | 4 | 4 |
| 122 | CT | 2 | 0 | 0 | 314 | CT | 4 | 4 | 4 |
| 123 | T | 2 | 0 | 0 | 315 | CT | 4 | 4 | 4 |
| 124 | T | 3 | 3 | 0 | 316 | CT | 4 | 4 | 4 |
| 125 | T | 3 | 2 | 2 | 317 | T | 4 | 4 | 0 |
| 126 | C | 2 | 2 | 0 | 318 | CT | 4 | 4 | 4 |
| 127 | C | 3 | 0 | 0 | 319 | CT | 2 | 3 | 3 |
| 128 | CT | 3 | 0 | 0 | 320 | CT | 3 | 4 | 0 |
| 129 | T | 2 | 0 | 0 | 321 | CT | 3 | 3 | 0 |
| 130 | T | 3 | 3 | 0 | 322 | CT | 1 | 4 | 3 |
| 131 | CT | 2 | 0 | 0 | 323 | CT | 1 | 2 | 1 |
| 132 | C | 3 | 0 | 0 | 324 | T | 1 | 1 | 0 |
| 133 | T | 2 | 0 | 0 | 325 | C | 1 | 1 | 2 |
| 134 | CT | 3 | 0 | 0 | 326 | CT | 2 | 2 | 2 |
| 135 | C | 3 | 0 | 0 | 327 | T | 2 | 2 | 3 |
| 136 | T | 3 | 0 | 0 | 328 | T | 2 | 2 | 2 |
| 137 | CT | 3 | 0 | 0 | 329 | CT | 2 | 2 | 3 |
| 138 | CT | 3 | 0 | 0 | 330 | C | 4 | 2 | 2 |
| 139 | CT | 2 | 0 | 0 | 331 | T | 2 | 4 | 0 |
| 140 | C | 2 | 0 | 0 | 332 | CT | 2 | 3 | 1 |
| 141 | CT | 3 | 2 | 0 | 333 | CT | 1 | 2 | 0 |
| 142 | T | 2 | 3 | 3 | 334 | T | 2 | 2 | 0 |
| 143 | T | 2 | 2 | 0 | 335 | CT | 2 | 2 | 0 |
| 144 | T | 1 | 2 | 0 | 336 | CT | 2 | 2 | 2 |
| 145 | T | 1 | 0 | 0 | 337 | T | 1 | 1 | 3 |
| 146 | T | 3 | 2 | 0 | 338 | T | 2 | 2 | 0 |
| 147 | CT | 3 | 0 | 0 | 339 | T | 2 | 3 | 0 |
| 148 | T | 2 | 1 | 0 | 340 | CT | 1 | 1 | 0 |
| 149 | T | 2 | 0 | 0 | 341 | T | 1 | 3 | 0 |
| 150 | T | 4 | 2 | 0 | 342 | CT | 1 | 2 | 0 |
| 151 | T | 3 | 2 | 0 | 343 | CT | 1 | 3 | 3 |
| 152 | T | 2 | 0 | 0 | 344 | T | 1 | 3 | 0 |
| 153 | T | 2 | 0 | 0 | 345 | CT | 2 | 2 | 0 |
| 154 | T | 2 | 0 | 0 | 346 | T | 2 | 3 | 0 |
| 155 | T | 2 | 0 | 0 | 347 | CT | 2 | 2 | 0 |
| 156 | CT | 2 | 0 | 0 | 348 | CT | 4 | 2 | 0 |
| 157 | CT | 2 | 2 | 0 | 349 | C | 1 | 1 | 2 |
| 158 | CT | 2 | 2 | 0 | 350 | C | 3 | 2 | 0 |
| 159 | CT | 3 | 1 | 0 | 351 | CT | 2 | 3 | 0 |
| 160 | C | 1 | 0 | 0 | 352 | T | 2 | 3 | 0 |
| 161 | T | 3 | 0 | 0 | 353 | T | 2 | 1 | 0 |
| 162 | CT | 3 | 3 | 0 | 354 | T | 2 | 2 | 0 |
| 163 | CT | 2 | 0 | 0 | 355 | T | 2 | 2 | 0 |
| 164 | T | 2 | 0 | 0 | 356 | CT | 4 | 4 | 0 |
| 165 | T | 1 | 0 | 0 | 357 | C | 2 | 0 | 0 |
| 166 | CT | 2 | 3 | 0 | 358 | T | 1 | 0 | 0 |
| 167 | CT | 3 | 4 | 4 | 359 | CT | 1 | 1 | 1 |
| 168 | CT | 3 | 3 | 4 | 360 | T | 2 | 2 | 2 |
| 169 | CT | 2 | 3 | 0 | 361 | CT | 3 | 3 | 0 |
| 170 | T | 2 | 0 | 0 | 362 | C | 1 | 3 | 2 |
| 171 | T | 2 | 2 | 2 | 363 | C | 1 | 3 | 2 |
| 172 | T | 3 | 3 | 0 | 364 | CT | 1 | 2 | 0 |
| 173 | CT | 3 | 3 | 0 | 365 | T | 1 | 1 | 0 |
| 174 | CT | 2 | 3 | 0 | 366 | T | 1 | 0 | 0 |
| 175 | T | 3 | 3 | 0 | 367 | CT | 3 | 1 | 0 |
| 176 | CT | 3 | 3 | 0 | 368 | T | 1 | 2 | 0 |
| 177 | T | 2 | 2 | 0 | 369 | CT | 2 | 1 | 0 |
| 178 | CT | 2 | 2 | 0 | 370 | T | 2 | 3 | 0 |
| 179 | CT | 3 | 3 | 0 | 371 | C | 1 | 2 | 0 |
| 180 | CT | 3 | 0 | 0 | 372 | CT | 2 | 4 | 0 |
| 181 | CT | 3 | 3 | 0 | 373 | T | 3 | 2 | 0 |
| 182 | CT | 2 | 0 | 0 | 374 | CT | 2 | 1 | 0 |
| 183 | T | 2 | 3 | 0 | 375 | CT | 1 | 0 | 0 |
| 184 | CT | 3 | 3 | 0 | 376 | CT | 2 | 2 | 3 |
| 185 | T | 3 | 0 | 0 | 377 | CT | 1 | 1 | 0 |
| 186 | T | 3 | 0 | 0 | 378 | CT | 2 | 3 | 3 |
| 187 | T | 2 | 3 | 3 | 379 | CT | 1 | 1 | 0 |
| 188 | T | 2 | 3 | 2 | 380 | T | 2 | 2 | 0 |
| 189 | T | 2 | 3 | 2 | 381 | T | 1 | 2 | 0 |
| 190 | CT | 1 | 2 | 0 | 382 | T | 4 | 3 | 3 |
| 191 | T | 3 | 3 | 0 | 383 | CT | 3 | 2 | 0 |
| 192 | T | 2 | 3 | 0 | 384 | T | 3 | 3 | 0 |
